# It Takes Two to Tango: Defining an Essential Second Active Site in Pyridoxal 5′-Phosphate Synthase

**DOI:** 10.1371/journal.pone.0016042

**Published:** 2011-01-21

**Authors:** Cyril Moccand, Markus Kaufmann, Teresa B. Fitzpatrick

**Affiliations:** 1 Department of Botany and Plant Biology, University of Geneva, Geneva, Switzerland; 2 Bio-Molecular Analysis Platform, University of Geneva, Geneva, Switzerland; University Paris Diderot-Paris 7, France

## Abstract

The prevalent *de novo* biosynthetic pathway of vitamin B6 involves only two enzymes (Pdx1 and Pdx2) that form an ornate multisubunit complex functioning as a glutamine amidotransferase. The synthase subunit, Pdx1, utilizes ribose 5-phosphate and glyceraldehyde 3-phosphate, as well as ammonia derived from the glutaminase activity of Pdx2 to directly form the cofactor vitamer, pyridoxal 5′-phosphate. Given the fact that a single enzyme performs the majority of the chemistry behind this reaction, a complicated mechanism is anticipated. Recently, the individual steps along the reaction co-ordinate are beginning to be unraveled. In particular, the binding of the pentose substrate and the first steps of the reaction have been elucidated but it is not known if the latter part of the chemistry, involving the triose sugar, takes place in the same or a disparate site. Here, we demonstrate through the use of enzyme assays, enzyme kinetics, and mutagenesis studies that indeed a second site is involved in binding the triose sugar and moreover, is the location of the final vitamin product, pyridoxal 5′-phosphate. Furthermore, we show that product release is triggered by the presence of a PLP-dependent enzyme. Finally, we provide evidence that a single arginine residue of the C terminus of Pdx1 is responsible for coordinating co-operativity in this elaborate protein machinery.

## Introduction

Vitamin B6 is essential for all organisms and has been shown to be involved in a diverse array of processes from the maintenance of the nervous and immune systems in animals and humans [1], to playing a role as an antioxidant in several different organisms [2]–[4] and more recently to potentially act as an antitumor agent [5]. It is a necessary cofactor in its form as pyridoxal 5′-phosphate (PLP), being required for the catalytic activity of over 140 metabolic enzymes. As a cofactor, it has been described as participating in transamination, decarboxylation, racemization, α,β-elimination and C_α_-C_β_ bond cleavage reactions [6]. Two routes to *de novo* vitamin B6 biosynthesis have been described, according to the enzymes and substrates used [7]. The less common route found in *Escherichia coli* and a few other members of the γ-division of proteobacteria that involves seven enzymes, is referred to as the DXP-dependent pathway [8], since it utilizes deoxyxylulose 5-phosphate (DXP) as a precursor to form the vitamin [9]–[11]. However, most organisms that can synthesize this vitamin, including fungi, plants, the majority of eubacteria and archaea, utilize a DXP-independent pathway, which remarkably involves only two enzymes, Pdx1 and Pdx2 [3], [12]–[14]. The latter two entities function as a glutamine amidotransferase that utilizes ribose 5-phosphate (R5P) and glyceraldehyde 3-phosphate (G3P) as well as glutamine to directly synthesize PLP [15], [16]. The complex of Pdx1 and Pdx2 displays a surprisingly ornate architecture, which in bacteria consists of 24 subunits; two hexameric rings of the synthase, Pdx1 (a (β/α)_8_-barrel), to which the glutaminase, Pdx2 (a three-layered αβα sandwich), attaches to each individual Pdx1 subunit [17], [18]. Pdx1 active site residues have been confirmed to be located at the C-terminal end of its (β/α)_8_-barrel, whereas the active site of Pdx2 is located at its interface with the N-terminal region of Pdx1 at the other end of the (β/α)_8_-barrel [17], [18]. The ammonia resulting from glutamine hydrolysis by Pdx2 is thought to be channeled in a putative tunnel to Pdx1 during the initial steps of pyridoxal 5′-phosphate formation [17]. While a comparison by X-ray crystallography of the Pdx1 oligomer with the Pdx1-Pdx2 complex as well as biochemical analyses revealed regions that become ordered upon complex formation [17]–[21], namely an extra N-terminal α-helix, αN, as well as an extra internal α-helix, α2′, the C terminus of Pdx1 consisting of 24 amino acids in *Bacillus subtilis* had eluded structural characterization, due to its inherent flexibility. Recently, it was shown to be required for catalysis, indeed mediating intersubunit cross-talk between two neighboring protomers of Pdx1, in addition to acting as a flexible lid that bridges and shields the active site of an adjacent subunit [21]. Moreover, the latter study showed that R5P triggers co-operativity within Pdx1, a feature coordinated by the C terminus, and furthermore that the affinity for the substrate is enhanced upon interaction with the Michaelis complex of Pdx2 and glutamine suggesting allostery. However, the key residue(s) involved in these molecular gymnastics remained to be elucidated.

As for other (β/α)_8_-barrel proteins, the active site located at the C-terminal end of the barrel in Pdx1 was implicit and has been shown by mass spectrometry and X-ray crystallography to accommodate the pentose phosphate substrate, R5P, and is referred to as P1 (for phosphate binding) [17]–[19], [22]. However, the accommodation of the second substrate, G3P, in addition to how the chemistry of the PLP biosynthesis reaction would be completed could not be accounted for. Interestingly, in the several structures of bacterial Pdx1's that have been determined so far, sulfate, chloride or phosphate ions were shown to bind to a second site, referred to as P2, located at the hexamer interface of the Pdx1 dodecamer [17]–[20]. The P2 site is made up from H115, R137, R138 in addition to K187 from α6′ of a subunit of the opposing hexamer (*B. subtilis* numbering). To date, this site has not been probed for functionality during the course of PLP biosynthesis. Intriguingly, while the isolated intermediates along the first-half of the reaction co-ordinate with the pentose phosphate substrate have been shown to be covalently attached to K81 in the P1 site, a second conserved lysine residue (K149), located between the P1 and P2 sites, albeit pointing towards the latter, has been demonstrated to be involved in PLP formation [16], [22], [23]. However, its molecular role has not been defined. Thus, given the above facts, there is precedence for a catalytic function within the P2 site.

Here, firstly, we dissect the functionality of the flexible C terminus of Pdx1 and show that a single arginine residue is fundamental for coordinating active site closure and co-operativity within Pdx1 itself, upon binding the pentose phosphate substrate. Moreover, the role of this residue, acts independently to the allostery imparted by Pdx2, which enhances the affinity for the substrate. Furthermore, we show that the P2 site is necessary for the transformation of the second substrate, G3P, into the product and is also where the completion of the synthesis of the PLP molecule takes place. Finally, we demonstrate that the PLP product remains covalently bound to K149, ratifying the role of this residue, and that release of the product is triggered by the presence of an enzyme dependent on PLP as a cofactor. The latter serves to regulate the release of the PLP molecule such that the expected non-specific chemical reactivity of this aldehyde can be controlled to prevent damage within the cellular milieu.

## Results

### A C-terminal arginine residue is essential for co-operativity observed in PLP synthase

**Figure 1:**
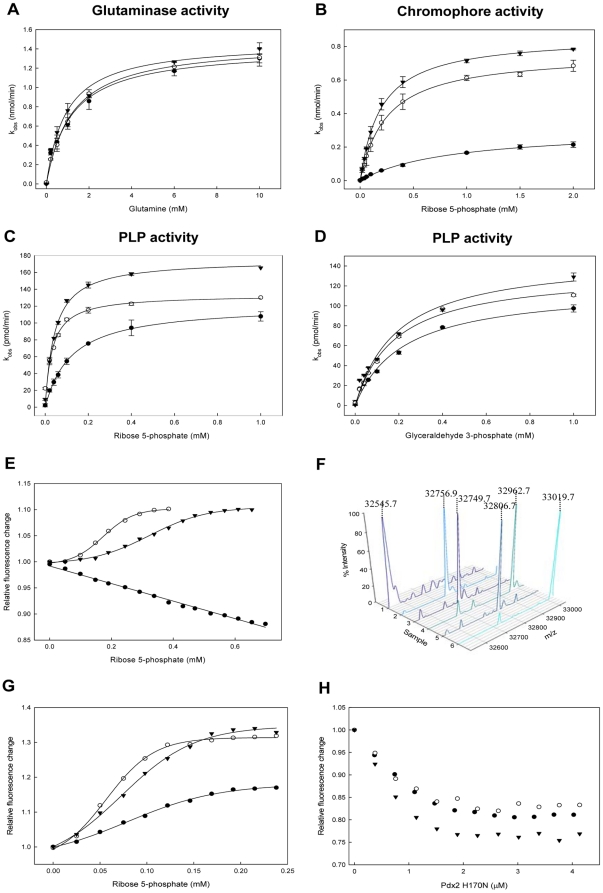
Arginine 288 is essential to co-ordinate the positive co-operative effect of ribose 5-phosphate in Pdx1. (A–D), Glutaminase, chromophore and PLP synthase activity as a function of glutamine (Gln), ribose 5-phospate (R5P) or glyceraldehyde 3-phosphate (G3P), respectively, of Pdx1 wild type (○), Pdx1 R288A (•) or Pdx1 R288K (▾). (E) Titration of either Pdx1 wild type (○), Pdx1 R288A (•) or Pdx1 R288K (▾) with increasing concentrations of R5P. A plot of the relative fluorescence change at 350 nm (excitation λ = 295 nm) *versus* the concentration of R5P demonstrates sigmoidal binding behavior for wild type and R288K, but not for R288A. (F) ESI-mass spectra of Pdx1 wild type (1–2), Pdx1 R288A (3–4) and Pdx1 R288K (5–6) in the absence (1,3,5) or presence (2,4,6) of R5P. The *m/z* difference of 212 Da in the presence of the substrate results from the binding of R5P to form a covalent adduct with Pdx1 [22]. (G) Titration of either preassembled PLP synthase (i.e. Pdx1, Pdx2 H170N and glutamine, [21]) with increasing concentrations of R5P. Preassembled PLP synthase exhibits sigmoidal binding behavior similar to that of Pdx1 alone but with a reduced K_0.5_ for R5P. (H) Relative fluorescence change of Pdx1 in the presence of increasing concentrations of Pdx2 H170N and added glutamine.

Given the recent demonstration that the flexible C-terminal region of Pdx1 is essential for catalysis [21], we sought to identify the key residue(s) in this region necessary for maintaining functionality. Of the residues in this part of the Pdx1 protein, we noted the strict conservation of an arginine residue (R288 *B. subtilis* numbering) at the very C terminus [21]. Site directed mutagenesis was utilized to probe its functionality by mutation to either an alanine or a lysine (R288A or R288K, respectively). Enzymatic activity was monitored in either of the three ways possible for PLP synthase, i.e. the ability to form the chromophoric intermediate along the reaction co-ordinate [22], the product PLP, or the capacity of Pdx1 to initiate glutaminase activity in its partner protein Pdx2 [15]. Both the Pdx1 R288A and R288K muteins were able to activate the glutaminase activity of Pdx2 to a similar extent as the wild type protein (Figure 1A and Table 1A). This indicates that such a mutation in the C-terminal region of Pdx1 does not affect the 3D fold of this enzyme, at least at the Pdx1-Pdx2 interface. However, the specificity constant for the formation of the chromophoric intermediate with Pdx1 R288A was estimated to be reduced 9-fold compared to wild type (k_cat_/K_M_ 0.09 min^−1^.M^−1^ and 0.8 min^−1^.M^−1^, respectively) as reflected in both k_cat_ and K_M_, whereas no significant change in the activity of the R288K mutein compared to wild type was noted (Figure 1B and Table 1A). Furthermore, while the k_cat_ for PLP formation by either mutant enzyme was not significantly affected compared to wild type enzyme, it could be seen that there was a significant increase in the K_M_ for R5P with the R288A mutein, while no significant change was observed for G3P (Figure 1C and D and Table 1A). Therefore, we postulated that R288 is, at least, necessary for the efficiency of Pdx1 with the R5P substrate and may be directly involved in its binding.

**Table 1:** Kinetic constants for the three measurable catalytic activities of PLP synthase: Pdx1 chromophore activity, PLP synthesis activity and Pdx2 glutaminase activity.

Table 1A: Summary of Pdx1 R288 mutein kinetic constants^a^					
	K_M_ R5P	k_cat_	k_cat_/K_M_		
Chromophore activity	(mM)	(min^−1^)	(min^−1^.mM^−1^)		
Pdx1 wild type	0.25±0.01	0.20±0.01	0.80		
R288K	0.20±0.01	0.22±0.01	1.10		
R288A	0.85±0.07	0.08±0.00	0.09		
	K_M_ R5P	K_M_ G3P	k_cat_	k_cat_/K_M_ R5P	k_cat_/K_M_ G3P
PLP activity	(mM)	(mM)	(min^−1^)	(min^−1^.mM^−1^)	(min^−1^.mM^−1^)
Pdx1 wild type	0.032±0.008	0.186±0.017	0.033±0.002	1.031	0.177
R288K	0.043±0.003	0.195±0.033	0.037±0.002	0.925	0.189
R288A	0.122±0.006	0.220±0.031	0.030±0.002	0.245	0.136
	K_M_ glutamine	k_cat_	k_cat_/K_M_		
Pdx2 glutaminase activity	(mM)	(min^−1^)	(min^−1^.mM^−1^)		
Pdx1 wild type	1.16±0.18	1.77±0.08	1.53		
R288K	0.93±0.15	1.84±0.08	1.97		
R288A	1.20±0.08	1.83±0.04	1.53		
^a^Assays were performed at 37°C in 50 mM Tris-Cl, pH 7.5 employing native (untagged) proteins					

Table 1B: Summary of Pdx1 P2 mutein kinetic constants^a^					
	K_M_ R5P	k_cat_	k_cat_/K_M_		
Chromophore activity	(mM)	(min^−1^)	(min^−1^.mM^−1^)		
Pdx1 wild type	0.20±0.01	0.042±0.001	0.21		
R138A	0.17±0.01	0.050±0.001	0.29		
H115A	0.19±0.03	0.032±0.002	0.17		
K187A	0.20±0.02	0.038±0.001	0.19		
H115A/R138A	0.16±0.02	0.033±0.008	0.21		
E105D	0.16±0.02	0.071±0.002	0.44		
	K_M_ R5P	K_M_ G3P	k_cat_	k_cat_/K_M_ R5P	k_cat_/K_M_ G3P
PLP activity	(mM)	(mM)	(min^−1^)	(min^−1^.mM^−1^)	(min^−1^.mM^−1^)
Pdx1 wild type	0.040±0.001	0.155±0.023	0.036±0.005	0.900	0.232
R138A	0.044±0.005	0.262±0.026	0.012±0.001	0.273	0.046
H115A	0.035±0.002	0.229±0.055	0.008±0.000	0.228	0.035
K187A	0.026±0.005	0.267±0.018	0.030±0.001	1.153	0.112
H115A/R138A	0.043±0.005	0.307±0.075	0.006±0.000	0.140	0.020
E105D	0.040±0.005	0.249±0.026	0.014±0.001	0.350	0.056
	K_M_ glutamine	k_cat_	k_cat_/K_M_		
Pdx2 glutaminase activity	(mM)	(min^−1^)	(min^−1^.mM^−1^)		
Pdx1 wild type	1.16±0.16	1.45±0.08	1.25		
H115A	1.33±0.18	1.32±0.05	1.00		
R138A	1.54±0.19	1.46±0.06	0.95		
K187A	1.31±0.15	1.42±0.05	1.08		
H115A/R138A	1.44±0.15	1.41±0.05	0.98		
E105D	1.12±0.14	1.58±0.06	1.41		
^a^Assays were performed at 37°C in 50 mM Tris-Cl, pH 7.5 employing hexahistidine tagged proteins					

Pdx1 wild type and various muteins are as indicated. R5P and G3P refer to ribose 5-phosphate and glyceraldehyde 3-phosphate, respectively.

In this context, we recently demonstrated binding of R5P to Pdx1 by fluorescence spectroscopy through the fortuitous presence of a single tryptophan residue at the very C terminus of the *B. subtilis* protein. Specifically, in the presence of the pentose phosphate substrate, there is an *increase* in the fluorescence emission maximum of Pdx1 at 350 nm [21]. Moreover, changes in the intrinsic protein fluorescence as a function of the pentose phosphate sugar concentration demonstrated co-operativity in Pdx1 upon binding this substrate, with a corresponding Hill coefficient of approximately 3.3 [21]. Here a comparison of the wild type enzyme with the two R288 muteins shows that co-operativity is completely abolished with the R288A mutant protein, whereas co-operativity is still observed with the lysine mutant (Figure 1E). However, while the Hill coefficient observed with Pdx1 R288K is similar to that of the wild type enzyme (3.2±0.3) there is a significant increase in K_0.5_ (348±13 µM) compared to the wild type enzyme (183±2 µM). As one of the first steps in PLP synthase catalysis is the covalent binding of the pentose phosphate sugar substrate to an active site lysine residue, manifested as an additional mass of 212 Da [16], [18], [22], we also made use of ESI-MS to establish if R5P indeed binds to the Pdx1 muteins. This analysis revealed that both Pdx1 mutants can form the R5P covalent adduct as for wild type under the same conditions (Figure 1F). Therefore, we can infer that R288 is necessary to communicate the cooperative response to the neighboring protomer to enhance the efficiency of transformation of the R5P substrate.

Furthermore, the Michaelis complex of Pdx2 and glutamine has an allosteric effect on Pdx1, observed as a decrease in K_0.5_ for R5P upon formation of the Pdx1:R5P:Pdx2:glutamine quaternary complex [21]. This can be demonstrated by incubating Pdx1 with the catalytically inert Pdx2 H170N mutein and glutamine all of which are known to assemble as a stable 1∶1 protein complex referred to as preassembled PLP synthase [17], and titrating with R5P. Here we assessed the effect of the R288A and R288K muteins on the allostery observed in Pdx1. As shown in Figure 1G, a significant reduction in K_0.5_ for R5P was observed with wild type Pdx1 in complex with Pdx2 H170N:glutamine compared to Pdx1 alone (62.4±2 µM and 183±2 µM, respectively) with a slightly reduced Hill coefficient for the preassembled PLP synthase complex (2.6±0.2) corroborating our earlier results [21]. Interestingly, in the presence of Pdx2 H170N and glutamine, the Pdx1 R288A mutein regains the ability to bind the pentose phosphate substrate in a co-operative manner. However, both the Hill coefficient (1.9±0.2) and K_0.5_ (114±10.8 µM) were impaired in comparison to the wild type enzyme. The behavior of the R288K mutant was also affected compared to the wild type enzyme (2.2±0.3 and 91.2±7 µM for the Hill coefficient and K_0.5_, respectively) but to a lesser extent than Pdx1 R288A (Figure 1G). The fluorescence spectroscopy approach can also be used to probe conformational changes upon interaction of Pdx1 with just the Michaelis complex of Pdx2 H170N and glutamine, i.e. in the absence of R5P, as demonstrated by Raschle *et al.* [21]. Specifically, titration of Pdx1 with Pdx2 H170N in the presence of glutamine leads to a *decrease* in fluorescence intensity as a function of the concentration of Pdx2 H170N and glutamine, demonstrating an allosteric effect of Pdx2:glutamine on the spatially separated C-terminal region of Pdx1. The analogous experiment here shows that the R288 muteins behave similar to the wild type Pdx1 enzyme (Figure 1H). As an additional control we also estimated the oligomeric state of the R288A mutein using size exclusion chromatography coupled to static light scattering. This experiment showed that Pdx1 R288A behaves similar to the wild type enzyme, i.e. it retains the ability to form the dodecamer (Figure S1A and B).

All of the above data demonstrates that the functionality of this conserved arginine residue is exclusive to the presence of R5P. As the C terminus is thought to bridge adjacent protomers and as we observed an affect on both R5P binding as well as catalysis, we tested the hypothesis that R288 could pose as an “arginine finger”. The latter is frequently observed in multimeric protein structures and plays either a structural or catalytic role [24], [25]. In its latter capacity the arginine finger has been shown to trigger phosphate hydrolysis in an adjacent protomer [26]. As one of the steps preceding chromophoric adduct formation in the biosynthesis of PLP is release of inorganic phosphate from R5P [22], we tested if R288 is necessary for this step in PLP synthase. However, no significant change was observed for either the R288A or R288K muteins compared to wild type (Table S1 and Figure S2). Thus, it must be inferred that R288 is mainly a structural determinant essential for R5P binding and co-operativity in Pdx1.

### Assigning functionality to the P2 site of Pdx1 in PLP synthesis

**Figure 2:**
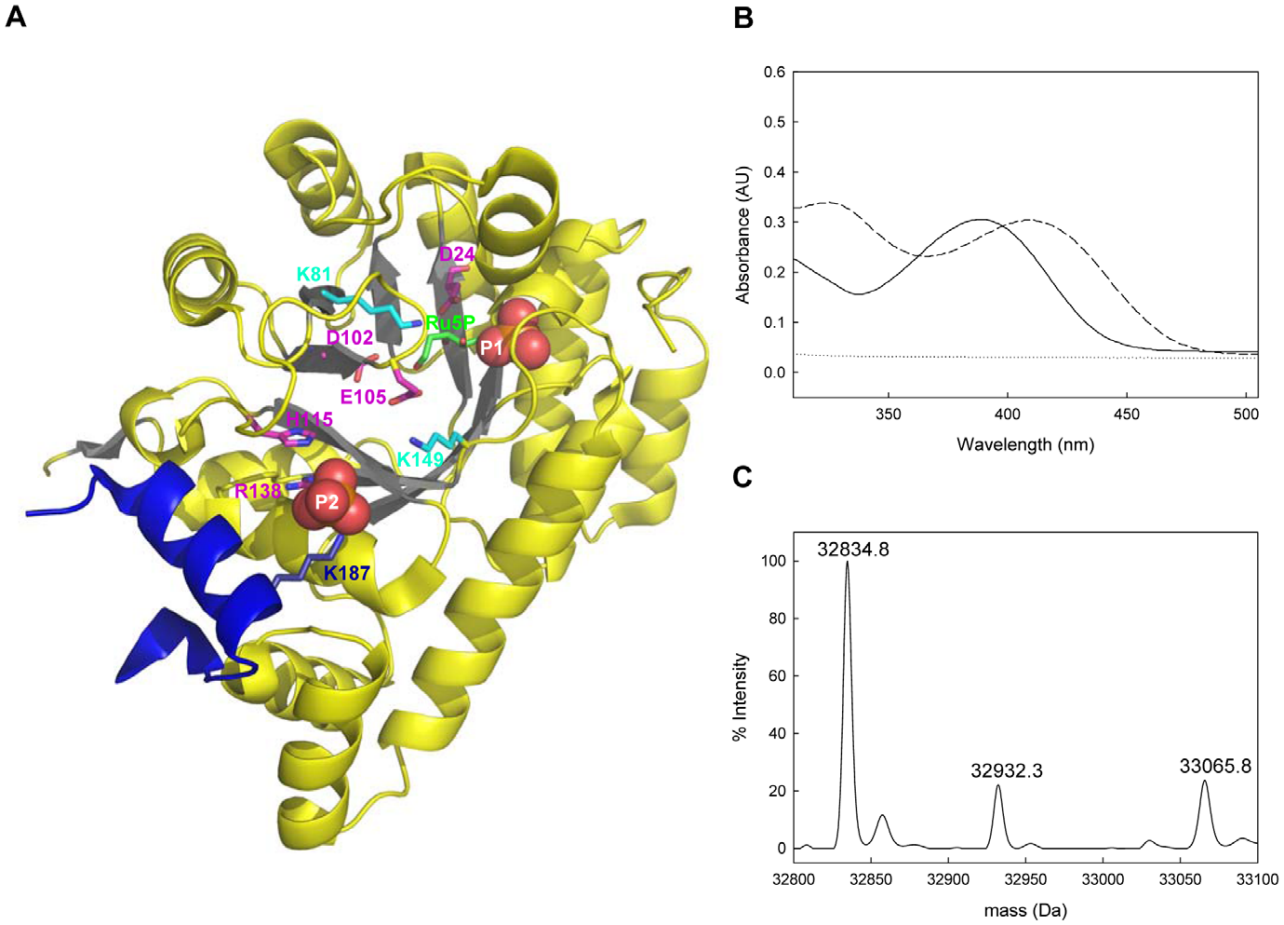
Substrate and product binding in Pdx1. (A) A single subunit of Pdx1 with ribulose 5-phosphate bound (Protein Data Bank code 2ISS). The P1 and P2 phosphate-binding sites are indicated as orange and red spheres, respectively. Catalytic and/or binding residues in the P2 site are highlighted. (B) Pdx1 covalently binds PLP. The *dashed* line represents Pdx1 wild type (100 µM) that had made PLP *de novo*. The solid line represents the previous sample treated with 0.1 M sodium hydroxide. The *dotted* line represents 20 mM HEPES buffer alone at pH 8 containing 150 mM sodium chloride. (C) ESI-MS analysis of Pdx1 wild type with reduced PLP. Reduction of the final product PLP on Pdx1 wild type reveals masses corresponding to the unmodified protein (32834.8 Da) and that with PLP bound (33065.8 Da; +231 Da), in addition to that corresponding to the protein with chromophoric intermediate covalently bound in the reduced form (32932.3 Da; +97 Da).

**Figure 3:**
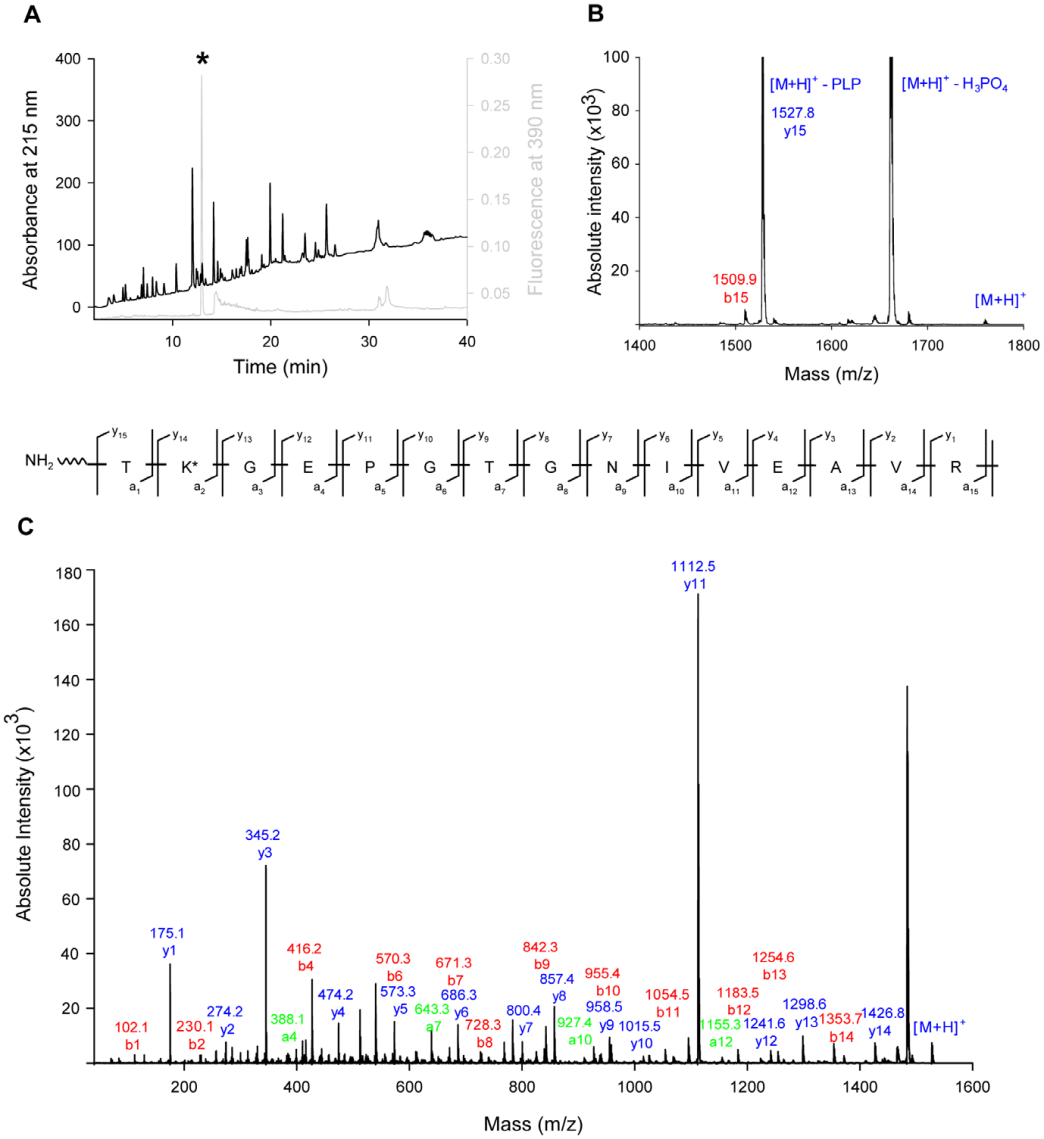
The final reaction steps of vitamin B6 biosynthesis take place at a second active site in Pdx1. (A) RP-HPLC elution profile of tryptic peptides of Pdx1 based on the absorbance at 215 nm, following PLP reconstitution and reduction to trap the final product. The gray line indicates the elution profile based on the fluorescence emission at 390 nm upon excitation at 320 nm. (B) MALDI-TOF/TOF analysis of the precursor ion *m/z = *1758.8 [M+H]^+^; a predominant loss of 98 Da corresponds to loss of phosphoric acid (*m/z = *1660.8 [M+H]^+^), while a further loss of 133 Da corresponds to loss of the pyridoxal moiety (*m/z = *1527.8 [M+H]^+^) matching the unmodified peptide. (C) MALDI-TOF/TOF analysis of the precursor ion *m/z* = 1527.8 [M+H]^+^, yielded a fragmentation pattern matching the predicted profile for the peptide TKGEPGTGNIVEAVR (residues 148–162). Observed β (red) and γ (blue) ions are indicated.

Two phosphate binding sites (referred to as P1 and P2) have been assigned to the synthase subunit, Pdx1, based on the co-crystallization of either pent(ul)ose phosphate sugar substrate or buffer inorganic anions, respectively, and as resolved in the X-ray structures of the Pdx1 proteins from *Geobacillus thermophilus*, *B. subtilis, Thermotoga maritima* and *Saccharomyces cerevisiae* [17]–[19] (Figure 2A). The Pdx1 P1 site has been unequivocally demonstrated to be the R(u)5P binding site [18] and the catalytic functionality of many of the residues in this area have been confirmed [17]. However, it is not known whether the entire chemistry of PLP biosynthesis takes place at Pdx1 P1 or if indeed the Pdx1 P2 site plays any role in this process. The most highly conserved residues surrounding this potential phosphate binding site are H115, R137, R138 in combination with K187 from a neighboring Pdx1 protomer of the adjacent hexamer (Figure 2A, *B*. *subtilis* numbering) [17]. In an attempt to assign functionality, we performed site directed mutagenesis of each of these residues to an alanine, in addition to constructing the double mutant H115A/R138A. The R137A mutation led to a misfolded version of the Pdx1 protein and was not pursued further (data not shown). The three enzymatic assays described earlier were used to characterize the remaining mutants with respect to their ability to form the chromophoric intermediate, the product PLP and to activate glutamine hydrolysis in Pdx2. All mutants were able to activate the glutaminase activity of Pdx2 (Table 1B) and retained the ability to form the R5P chromophoric intermediate with similar catalytic parameters to wild type Pdx1 (Table 1B). It is noteworthy that the C-terminally hexahistidine tagged versions of the Pdx1 wild type and P2 muteins (Table 1B) have slightly impaired catalytic activity for the formation of the chromophore compared to the native enzymes used to probe functionality of R288 (Table 1A). However, clear catalytic perturbations were observed for the formation of the product PLP (Table 1B). PLP formation was estimated to be 33%, 22% and 17% of the wild type activity for R138A, H115A and the H115A/R138A double mutant, respectively, while the activity of K187A was shown to be 84% of the wild type activity. The dramatic reduction in catalytic rate of R138A, H115A and the corresponding double mutant was mainly manifested in the k_cat_, severely impairing substrate specificity and demonstrating that these residues have a catalytic role to play in PLP formation. Interestingly, mutations in the Pdx1 P2 site consistently increased the K_M_ for G3P (up to 2-fold), while no corresponding effect was observed in the K_M_ for R5P (Table 1B). Moreover, ESI-MS as described above, confirmed that all muteins could bind R5P (data not shown). Given the bias of the Pdx1 P2 muteins towards the later steps in PLP formation, i.e. post chromophoric intermediate formation, and in particular the effect on G3P binding, we decided to probe product formation further. Two points are important to note in this context; firstly, the major rate-limiting step along the PLP biosynthesis reaction co-ordinate is in fact dissociation of the product [27] and secondly, the maximum absorbance for the formation of the product is at 414 nm in different buffers examined (i.e. phosphate, HEPES) at pH 8.0. This indicates that PLP is bound in a Schiff's base to Pdx1 (similar to enzymes dependent on it as a cofactor [28]), as free PLP, on the other hand, has an absorbance maximum at 388 nm [29]. Indeed, when we treated Pdx1 *de novo* synthesized PLP with 0.1 M sodium hydroxide there was a hypsochromic shift in the absorbance spectrum to a maximum at 388 nm (Figure 2B). This behavior is typical for PLP bound in a Schiff's base to a lysine residue in a protein [29]. Additionally, ESI-mass spectrometry analysis showed that wild type Pdx1 harboring PLP generated *de novo* followed by reduction with sodium borohydride displays an additional mass 231 Da above that of the observed mass of the apoprotein (32834.8 Da), and corresponds to a PLP-conjugated lysine (Figure 2C). Note, the additional peak observed at 32932.3 Da (+97 Da) in this spectrum is most likely due to the presence of residual Pdx1 with the covalently bound chromophoric intermediate in a reduced state [22]. These latter observations demonstrate that PLP is covalently bound to Pdx1 and prompted us to investigate its location within the protein. To identify the binding site of PLP, a single turnover of the biosynthesis reaction was reconstituted by incubating the wild type enzyme with the substrates, R5P and G3P while substituting Pdx2 and glutamine for ammonium sulfate. As for many other glutamine amidotransferases, an external source of ammonia (e.g. ammonium sulfate) can substitute for that provided by glutamine in the presence of the glutaminase domain [15]. As above, the final reaction intermediate was trapped by reduction with sodium borohydride and the resulting protein was subjected to digestion with trypsin. A direct analysis of the resulting peptides by MALDI-TOF mass spectrometry did not reveal any masses corresponding to an expected peptide plus a bound PLP molecule (data not shown). However, reduction of a Schiff's base between PLP and a residue within a peptide facilitates its isolation by RP-HPLC coupled to a fluorescence detector, as such peptides have an intrinsic fluorescence at 390 nm [30]. The elution profile of sodium borohydride treated Pdx1 revealed the appearance of a prominent new fluorescence peak in the reduced sample (Figure 3A). Analysis by MALDI-TOF mass spectrometry indicated the mass of an expected peptide within Pdx1, namely TKGEPGTGNIVEAVR (expected mass 1527.8 Da) with a reduced PLP molecule bound (expected mass 1758.8 Da). Indeed, in addition to the precursor ion of *m/z* = 1758.8 Da, we predominantly observed bands at an *m/z* of 1660 and 1527.8, corresponding to a loss of phosphoric acid (98 Da) and the pyridoxal moiety (133 Da), respectively (Figure 3B). A MALDI-TOF/TOF analysis of all three precursor ions yielded the sequence TKGEPGTGNIVEAVR (Figure 3C). As there is only a single lysine residue within this peptide, corresponding to K149, this demonstrates that the final intermediate in the biosynthesis of the PLP molecule must be in a Schiff's base with K149 in Pdx1. Given that covalent intermediates have been identified at K81 in P1 [18], [22] and now K149 in P2, it must be deduced that there is transfer of an intermediate along the reaction co-ordinate from the Pdx1 P1 to the Pdx1 P2 site. We noted the presence of a highly conserved glutamate residue (E105) that is located between K81 and K149 (Figure 2A). Site directed mutagenesis was utilized to probe changes in activity upon mutation to either an alanine or an aspartate (E105A or E105D, respectively). However, as the hydrolysis of glutamine is impaired for E105A, indicating a change in folding of this mutant and even though the chromophoric adduct can be formed but no PLP activity is detected with this mutation (data not shown), we will refrain from including this data in a functional hypothesis for this residue. Nevertheless, the Pdx1 E105D mutein was able to activate glutaminase activity to a similar extent as the wild type protein (Table 1B). Interestingly, the specificity constant for the formation of the chromophoric intermediate with Pdx1 E105D was estimated to be increased 2-fold compared to wild type (k_cat_/K_M_ 0.44 min^−1^.M^−1^ and 0.21 min^−1^.M^−1^, respectively). Indeed, while the k_cat_ for PLP formation of this mutant enzyme was significantly decreased compared to wild type, it could be seen that there was also a significant increase in the K_M_ for G3P with the E105D mutein, while no significant change was observed for R5P (Table 1B). This data suggests that this residue may provide a “steric gate” in the transfer of an intermediate between the Pdx1 P1 and P2 sites.

### Product release is triggered by an enzyme dependent on it as a cofactor

**Figure 4:**
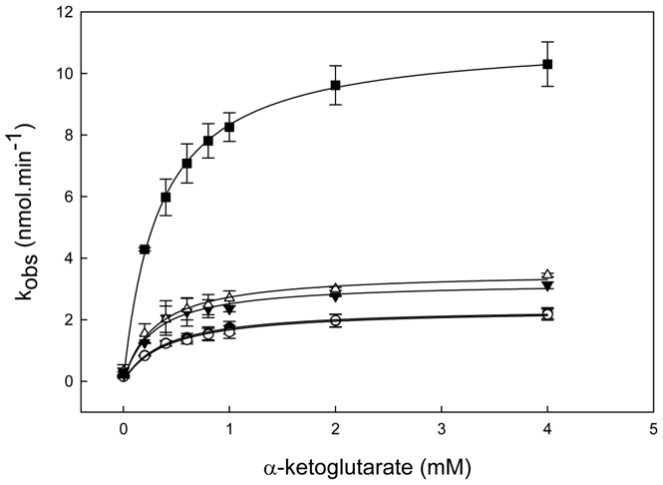
Steady state kinetics of *Bs*AAT2. Asparate aminotransferase activity of *Bs*AAT2 (4 µM) as isolated (○), *Bs*AAT2 (4 µM) + Pdx1 wild type that had made PLP *de novo* (4 µM of PLP) (▪), *Bs*AAT2 + apoPdx1 (▾), *Bs*AAT2 + Pdx1 with sodium borohydride reduced PLP (▵), *Bs*AAT2 + Pdx2 (•). The decrease in absorbance at 340 nm upon oxidation of NADH during the process of oxaloacetate oxidation to malate facilitated by malate dehydrogenase is shown as a function of the concentration of α-ketoglutarate. Assays were carried out in 30 mM potassium phosphate buffer, pH 7.5, and at 25°C employing *Bs*AAT2 as isolated, malate dehydrogenase (0.25 mg/ml), NADH (0.2 mM), aspartate (20 mM), Pdx1 wild type-PLP (4 µM of PLP), apoPdx1 wild type (4 µM), Pdx1-reduced PLP (4 µM) and Pdx2 (4 µM). The concentration of PLP bound to Pdx1 was estimated using an extinction coefficient of 5380 M^−1^.cm^−1^.

We next attempted to explain the observation that the final reaction product PLP is tightly bound to the enzyme [27], [31] in a more physiological context. As stated above, this represents a rate-limiting step in the overall biosynthesis reaction. We hypothesized that the retention of the product by the enzyme serves to regulate the release of the PLP molecule such that the expected non-specific chemical reactivity of this aldehyde can be controlled to prevent damage within the cellular milieu. In this context, release of the PLP molecule would be anticipated in the presence of an enzyme dependent on PLP as a cofactor. To probe this hypothesis we cloned, expressed and purified a putative aspartate aminotransferase from *B. subtilis* (annotated as *AAT2*) that would be expected to use PLP as a cofactor. Activity measurements confirmed that *Bs*AAT2 catalyses the formation of L-glutamate and oxaloacetate from L-aspartate and α-ketoglutarate in a PLP-dependent manner (Figure 4 and Table 2). Notably, the activity of *Bs*AAT2 as isolated increased 5-fold in the presence of a stoichiometric concentration of PLP generated *de novo* from a single turnover reaction in Pdx1. This implies that the *Bs*AAT2 as isolated was not saturated with PLP. Importantly, the activity did not increase in the presence of apoPdx1, Pdx1 where the PLP had been irreversibly reduced with sodium borohydride, or Pdx2, which does not contain PLP (Figure 4 and Table 2). These results suggest that the PLP-dependent enzyme AAT2 from *B. subtilis* is able to trigger hydrolysis and release of PLP from Pdx1, allowing transfer of this highly reactive co-factor to the PLP-dependent enzyme. Indeed, we could no longer detect the formation of PLP by Pdx1 in the presence of AAT2 under these steady state conditions, indicating that the transfer is immediate (data not shown).

**Table 2:** Summary of AAT2 kinetic constantsa.

AAT2 activity	K_M_ α-ketoglutarate	k_cat_	k_cat_/K_M_
	(mM)	(min^−1^)	(min^−1^.mM^−1^)
AAT2	0.40±0.05	2.9±0.1	7.3
AAT2 + Pdx1:PLP	0.34±0.02	14.0±0.2	41.2
AAT2 + apoPdx1	0.29±0.05	4.0±0.2	13.8
AAT2 + Pdx1:reduced PLP	0.30±0.05	4.4±0.2	14.7
AAT2 + Pdx2	0.38±0.05	3.0±0.1	7.9

a Assays were performed at 25°C in 50 mM potassium phosphate containing 50 mM potassium chloride at pH 7.5.

## Discussion

The PLP synthase complex appears to be exceptional in its intrinsic multiple catalytic abilities, ranging from its presumed pentose and triose isomerizations to ammonia addition and aromatic ring formation in a single enzymatic system. Several of the mechanistic steps remain unclear and additional information on the mechanism of such a multifunctional enzyme is of great interest considering its potential as a drug target, being essential for survival of several micro-organisms and plants [32], [33], while not being found in humans. Indeed, several pathogenic microorganisms employ the DXP-independent pathway including *Corynebacterium diphteriae, Mycobacterium leprae* and *Plasmodium falciparum*, the latter being involved in the most severe form of malaria. Most recently, the pathway has been shown to be essential for the survival of *Mycobacterium tuberculosis*, a human pathogen the infection from which claims close to 2 million lives annually [34]. The high conservation of Pdx1 among organisms [3] together with the structural characterization of PLP synthase complexes from *B. subtilis* [17], *T. maritima* [18], *G. thermophilus* [19] and *S. cerevisiae* [20] enabled us to select amino acid residues potentially involved in catalysis of vitamin B6 biosynthesis. Several studies have already resolved steps in the early stage of biosynthesis of the vitamin [18], [22], [31], [35], [36]. Specifically, the reaction begins with the binding of the pentose phosphate substrate (most likely in its ring form). Opening of the furanose ring ensues and a covalent adduct develops between the sugar and an active site lysine residue in Pdx1 (K81 in *B. subtilis*) [18], [22]. In the presence of the active Michealis complex of Pdx2 and glutamine, this is followed by the loss of inorganic phosphate and water, leading to a highly conjugated arrangement that is chromophoric and is characterized by an absorbance maximum at 315 nm [22]. However, the remaining steps that occur upon G3P binding had not been explored even though several plausible mechanistic versions had been put forward [22], [31], [35], [36]. Moreover, it had been noted that although several residues at the P1 site in Pdx1 were confirmed to be involved in catalysis, they did not suffice to map the entire catalytic co-ordinate [17], [22]. Here we contribute several observations to unraveling and confirming the remaining steps of PLP biosynthesis. Firstly, we show that a conserved residue in the flexible C-terminal region is not only necessary for formation of the chromophoric intermediate in Pdx1 but is fundamental for the positive co-operativity observed within the ornate PLP synthase complex. As we have recently provided evidence that the C terminus carries out its function on a neighboring subunit [21], we hypothesize that this residue could function analogous to an ‘arginine finger’. Classically, an arginine finger is recognized as an arginine residue located on an adjacent subunit that can coordinate the phosphate group of a bound substrate, stabilize the transition state during phosphate hydrolysis and/or act as a trigger for conformational changes after hydrolysis [26]. Given that we observed a strong defect in R5P binding with the R288A Pdx1 mutant and no significant change in the release of inorganic phosphate (Table S1 and Figure S2), the most likely role for this residue is co-ordination of the phosphate group of R5P in the P1 site of an adjacent Pdx1 subunit. This hypothesis is corroborated by the fluorescence spectroscopy experiments, which demonstrate that the R288A mutation completely abolishes the co-operativity observed with R5P in Pdx1. Yet, we cannot rule out that this arginine residue may be involved in the proton shuffling that occurs on the way to forming the chromophoric intermediate. However, arginine residues are generally protonated at physiological pH and therefore considered poor candidates for the role of general bases. Nevertheless, they have emerged as bases in several enzymes [37]. In such a reaction, the arginine is activated by a carboxylate group. In Pdx1, the catalytic role of D24 has been confirmed [17], but the exact molecular nature of this role remains unclear. In this context, it could act as an activator of R288 resulting in proton shuffling at C5 prior to phosphate elimination as previously reported [35]. A tool for protein pK_a_ prediction of amino acids based on empirical relationships between protein pK_a_ shifts and structures called PROPKA [38] was used to give insights into the nature of these residues. An average of the calculated pK_a_'s of D24 from the Pdx1 dodecamer of *B. subtilis* (Protein Data Bank code 2NV2) gave a value of 4.7±0.3.

Although, we show that the R288A mutant does not permit co-operativity in Pdx1 alone, positive co-operativity is restored in the presence of Pdx2 and glutamine (Figure 1G). Given the dependence of co-operativity in this case on the preassembled PLP synthase complex, it is worth noting structural features that are specific to this complex but are absent in Pdx1 alone. Among the most relevant is the formation of helix, α2′, above the R5P binding site in the PLP synthase complex in the absence of substrate [17] and the observation that in the presence of R5P, both α2′ and α8′ in the PLP synthase complex move to tighten the Pdx1 P1 active site [21]. In the latter study, it was suggested that the movement of both α2′ and α8′, would additionally facilitate the movement of the Pdx1 C terminus (not seen in the crystal structures) to a less exposed environment, thus completing the closing of the active site albeit of the adjacent protomer [21]. Thus, the positive co-operativity observed for the Pdx1 R288A:R5P:Pdx2:glutamine assembled complex can be said to be exclusively derived from the presence of Pdx2 and glutamine. In other words, the R288 mutation “uncouples” the co-operativity imparted on the one hand by Pdx1 itself and on the other hand, that due to the Michaelis complex of Pdx2 and glutamine upon binding R5P. This opens the way to unravel the allosteric relay imparted by Pdx2 and glutamine on the remote Pdx1 C terminus. Indeed, the complete absence of co-operativity observed with the Pdx1 R288A mutant alone in the presence of R5P suggests that the R288 residue is instrumental in “sensing” the presence of the substrate. If this arginine residue is to additionally act in a catalytic capacity during the first steps of the reaction, i.e. before formation of the chromophoric intermediate its role could involve two steps, either: i) bestow rigidity to the C terminus by stabilizing the binding of the pentose phosphate substrate and thus permitting closure of the active site and/or ii) perform proton abstraction after activation by D24 as postulated above. Indeed, a role for an arginine and an aspartate residue in both domain closure and catalysis has already been reported for aspartate transcarbamylase from *E. coli* [39].

One of the most pertinent questions on the mechanism of PLP synthase pertains to the functionality of the second phosphate-binding site, P2, in Pdx1. Site-directed mutagenesis of either H115A or R138A within this site revealed a substantial effect on the formation of PLP, demonstrating that these residues are essential for catalysis. In particular, while the kinetic constants for chromophoric intermediate formation with the Pdx1 H115A, R138A or the corresponding double muteins are similar to those of the wild type enzyme, those for PLP formation are significantly reduced. Notably, there is a significant increase in the K_M_ of G3P with the mutant proteins in comparison to the wild type enzyme indicating impaired binding of this substrate. As Pdx1 shares the canonical triose phosphate isomerase fold (i.e. TIM barrel or (β/α)_8_ barrel) and the latter enzyme catalyses the reversible interconversion of dihydroxyacetone phosphate (DHAP) and D-glyceraldehyde 3-phosphate, we deemed a direct comparison with the active site of this enzyme appropriate. We have previously shown that Pdx1 can utilize both G3P and DHAP as substrates but has a strong preference for the former [15]. Thus, although complete triose isomerization has not been demonstrated for Pdx1, it could utilize H115 to donate a proton to the triose sugar for the formation of an enediol intermediate that is known to occur in the TIM reaction. Moreover, making use of the PROPKA software [38] a pK_a_ of 5.75±0.2 was calculated for H115 in Pdx1, which would support an acidic role for this residue. However, no other residues analogous to the TIM active site are present in the P2 site of Pdx1 with the exception of K187 that projects into the site from a neighboring protomer on the opposing hexameric ring (Figure 2A). Indeed, while the K187A mutant shows a slight reduction of the catalytic activity for PLP formation (Table 1B), it has a similar increase in K_M_ for binding G3P compared to the other Pdx1 P2 muteins suggesting that this residue is more involved in binding the triose phosphate substrate than in catalysis. However, we observed that the Pdx1 K187A mutein is disrupted in the dodecamer:hexamer equilibrium compared to wild-type under the same conditions and shows a stronger tendency towards hexamer rather than dodecamer (Figure S1C). As K187 is projecting into the Pdx1 P2 site from a protomer on a neighboring hexamer and is therefore important in maintaining the dodecameric state, this may be an alternative explanation for the observed decrease in the affinity for G3P. Nevertheless, the unequivocal demonstration that PLP is in a Schiff's base with K149 not only establishes a role for this residue but moreover corroborates that the Pdx1 P2 site is necessary for biosynthesis of the vitamin. While, mutagenesis of K149 showed that it had a catalytic role to play during PLP biosynthesis [22], the exact molecular role of this lysine residue has been a matter of controversy in the literature. This residue was originally thought to be the site of the pentose sugar substrate binding [16] but later both mass spectrometric [22] and X-ray structural work [18] disproved this idea, showing that K81 in P1 was the site of binding of R5P. The presence of the second active site (P2) in Pdx1 involved in the latter half of the reaction co-ordinate requires the transfer of a reaction intermediate from the P1 to the P2 site. While both sites are separated by 21 Å, the structural analyses have revealed a shallow groove lined with charged residues that connects Pdx1 P1 and P2 [17]. Moreover, the catalytic impairment of the conserved residue, E105, which is located between K81 and K149 indicates that it may play a role in this process. It remains to be established how and when the relocation from K81 to K149 occurs. It has been proposed that K149 could be conceived to undergo a side chain rotation to project into the Pdx1 P1 site [18], [19] instead of P2 as is observed in all of the structures determined so far. If this were to occur the NZ atom of K149 would be able to approach C5 of the pentose sugar substrate. However, with all of the data so far there is no obvious way to explain what would trigger this “swinging arm” mechanism [18], [19] of K149 from Pdx1 P2 to P1 and back to P2. Thus, while it is clear that P1 and P2 must co-operate and co-ordinate their movements with each other to affect the production of the PLP molecule the details of this dynamic “Tango” remain to be defined.

The final question we addressed was why the product remains tightly bound to PLP synthase. Our data clearly demonstrate that an enzyme dependent on the PLP product as a cofactor is capable of removing it from Pdx1 and utilizing it for catalytic activity. This represents an additional level of regulation for the PLP synthase complex and is of grave physiological importance as it ensures that the reactive aldehyde product is not released into the cellular milieu, where it could cause damage by reacting non-specifically with exposed lysine residues in proteins not dependent on PLP as a cofactor or complexing with free amino acids. As a corollary this implies that PLP synthase is primed for donating the product of its reaction and only does so when required and furthermore suggests that the ornate dodecameric architecture could serve as a storage form of the vitamin. Interestingly, pyridoxine oxidase, an enzyme of the salvage pathway that is present in all organisms, also retains the product PLP in a tight binding complex [40]. Therefore, we propose that the mechanism of PLP transfer can be generalized to all PLP dependent enzymes and opens an area for future investigation. Notably, enzymes catalyzing the formation of any of the other vitamers of B6, e.g. pyridoxol and pyridoxamine and their phosphorylated derivatives do not bind the products of their reactions tightly [10], [41]. Given the importance of this vitamin in nature, the work described here provides insight into yet another aspect of the fascinating control the cell imposes on itself in order to curtail possible points of damage and maintain functionality.

*While this manuscript was undergoing completion, we learned that the X-ray structure of PLP synthase from S. cerevisiae with PLP and G3P bound was accepted for publication [*42*] *. This article apparently confirms the presence of PLP in the P2 site, although they did not comment on if it was in a Schiff's base with K149. Interestingly, G3P was reported to bind in a third active site, based on co-crystallization of the apo-form with 10 mM dihydroxyacetone phosphate. However, neither the structures nor any kinetic data of implicated residues were available yet to judge this conclusion.*

## Materials and Methods

### Protein expression and purification

The constructs pET*Bs*Pdx1 and pET*Bs*Pdx2-His_6_ described by Raschle et al. (2005), in addition to pET*Bs*Pdx1-His_6_ and Pdx2-His_6_ H170N described by Strohmeier et al. (2006) were used in this study. The construct pET*Bs*AAT2 was generated by amplification of the *B. subtilis AAT2* gene with primers CTAGCTAGCATGGAAATAACACCGTCCGATGTC and CCGCTCGAGGCGGGATGTTTCTT-GTAATGACC that incorporate an NheI and XhoI restriction site at the 5′ and 3′ ends, respectively, for cloning into of pET21a (EMD Biosciences) such that when expressed a hexahistidine tag would be included at the C terminus. Protein expression and purification, for both the native and hexahistidine tagged proteins, were carried out as described by Raschle et al. (2005) and monitored by SDS-PAGE on either 12.5 or 15% polyacrylamide gels and staining with Coomassie Blue. Protein concentration was determined by the method of Bradford using bovine serum albumin as a standard [43].

### Site directed mutagenesis

Site directed mutagenesis of Pdx1 for single and double mutants was carried out using the QuikChange® XL Site Directed Mutagenesis Kit (Stratagene). The oligonucleotides used for mutagenesis are listed in Table S2.

### Enzyme assays

Distinct enzyme activities of PLP synthase can be separately measured as previously described [15], [22], namely the rate of glutamine hydrolysis by Pdx2, the formation of the end product PLP, the formation of the chromophoric reaction intermediate and the release of inorganic phosphate. All of these enzyme activities are detectable by UV-visible spectrophotometry. Glutaminase activity of Pdx2 was monitored by a coupled enzyme assay as described by Raschle et al. (2005). PLP formation was monitored by the change in absorbance at 414 nm in the presence of R5P, G3P, Pdx1 and glutamine, as described by Raschle et al. (2005, 2007). Formation of the chromophoric reaction intermediate was determined by the increase in absorbance at 315 nm in the presence of Pdx1, Pdx2, R5P, and glutamine [15]. Measurement of the release of inorganic phosphate was monitored by a coupled enzyme assay (EnzCheck® phosphate assay kit, Molecular Probes) as described by Raschle et al. (2007). AAT2 activity was determined via a coupled reaction with malate dehydrogenase as previously reported [44]. In this assay, the enzymatic conversion of L-aspartate and α-ketoglutarate to L-glutamate and oxaloacetate by AAT2 was measured. The decrease in absorbance at 340 nm upon oxidation of NADH during the process of oxaloacetate oxidation to malate facilitated by malate dehydrogenase is shown as a function of the concentration of α-ketoglutarate. Assays were carried out in 30 mM potassium phosphate buffer, pH 7.5, and at 25°C employing *Bs*AAT2 (4 µM), malate dehydrogenase (0.25 mg/ml), NADH (0.2 mM), aspartate (20 mM), Pdx1 that had made PLP *de novo* (4 µM of PLP), apoPdx1 (4 µM), Pdx1-reduced *de novo* synthesized PLP (4 µM) and Pdx2 ( µM). The concentration of PLP bound to Pdx1 was estimated using an extinction coefficient of 5380 M^−1^.cm^−1^.

### Equilibrium binding monitored by intrinsic protein fluorescence

The steady-state fluorescence measurements were conducted on a QuantaMaster 4 spectrofluorometer (Photon Technology International, Seefeld, Germany) as described previously [21]. The steady state kinetics for ribose 5-phosphate binding displayed sigmoidal behavior and were thus determined by nonlinear curve fitting to the Hill equation:
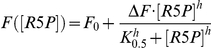



Where *F*([R5P]) represents the fluorescence signal at a given R5P concentration, *F_0_* is the minimal fluorescence signal, Δ*F* is the maximal change in fluorescence intensity, K_0.5_ is the R5P concentration at half-maximal fluorescence signal, and *h* is the Hill coefficient.

### Size exclusion chromatography and multi-angle light scattering (MALS) experiments

Native wild type or R288A Pdx1 as well as the hexahistidine tagged Pdx1 K187A (100 µl at 1 mg/ml) were first separated by size exclusion chromatography on a Superdex 200 10/300 GL column (GE Healthcare). The elution buffer was 50 mM potassium phosphate, pH 7.5, containing 50 mM potassium chloride and 0.01% sodium azide. A miniDAWN TREOS light scattering instrument (Wyatt Technologies, USA) was connected immediately downstream of the separation media for light scattering analysis. The data were analyzed using the software ASTRA (Wyatt Technologies, USA). An absorption coefficient of 368.0 (ml.g^−1^.cm^−1^) for *Bs*Pdx1 wild type, 369.0 (ml.g^−1^.cm^−1^) for Pdx1 R288A and 353.5 (ml.g^−1^.cm^−1^) for the Pdx1 K187A enzyme was used for the calculation of the weight average molecular mass (Mw).

### Identification of the peptide containing PLP

Isolated Pdx1 wild type was incubated with 1 mM DL-G3P, 1 mM R5P and 10 mM ammonium sulfate for 3 h at room temperature to generate PLP. Reductive fixation of PLP to the enzyme was performed by treatment with 4 mM sodium borohydride at 4°C. The resulting enzyme (1–1.2mg) was treated with 10 µg of trypsin (Sequencing Grade Modified Trypsin, Promega) in 50 mM Tris-Cl pH 7.5 at 37°C overnight. Digestion was terminated by the addition of formic acid to a final concentration of 2.7%, and the sample was centrifuged at 15′000 *g* for 30 min. The supernatant was applied to a C18 reversed-phase column XDB (1.8 µm, 4.6×50 mm, Agilent) equipped with a UV detector to monitor absorption at 215 nm and a fluorescence detector to monitor the reduced pyridoxal 5′-phosphate probe (excitation at 320 nm and emission at 390 nm). A gradient of 0–60% acetonitrile/1% trifluoroacetic acid (v/v) at 1 ml.min^−1^ was established over 45 min, held for 5 min, raised to 80% acetonitrile/1% trifluoroacetic acid (v/v) over 10 min, and then returned to the starting conditions over 15 min. Fractions of interest were analyzed by MALDI-TOF/TOF mass spectrometry (Bruker Ultraflex II).

## Supporting Information

Figure S1 **Size exclusion chromatography coupled to static light scattering analysis of Pdx1 wild type, R288A and K187A.**
Prior to multi-angle light scattering (MALS) measurements, size exclusion chromatography (SEC) was performed using a Superdex 200 10/300 column in 50 mM potassium phosphate buffer, pH 7.5, containing 50 mM potassium chloride using 1 mg/ml of enzyme. The combination of SEC and MALS reveal a predominant peak corresponding to the dodecameric form of Pdx1 (A) wild type or (B) Pdx1 R288A whereas (C) Pdx1K187A exhibits a dodecamer:hexamer equilibrium. The weight average molecular mass Mw mass (*black line*) and the Rayleigh ratio (*gray line*) were plotted in an elution volume dependent manner.
(TIF)

Figure S2 **Steady state kinetics for the release of inorganic phosphate from Pdx1 wild type (○), R288A (•) or R288K (▾).**
The rate of inorganic phosphate release was monitored employing a coupled enzymatic assay. The reaction was carried out in 50 mM Tris-Cl pH 7.5, containing 20 mM magnesium chloride, employing Pdx1 wild type or muteins (20 µM), Pdx2 (20 µM), glutamine (10 mM), 2-amino-6-mercapto-7-methyl-purine (0.2 mM) and purine nucleoside phosphorylase (1.5 U) as a function of the concentration of R5P.
(TIF)

Table S1 (DOC)

Table S2 (DOC)
