# Supplementary figures and images for: It Takes Two to Tango: Defining an Essential Second Active Site in Pyridoxal 5′-Phosphate Synthase
Source: PLoS One. 2011 Jan 21;6(1):e16042. doi: 10.1371/journal.pone.0016042 (PMC3024981; doi:10.1371/journal.pone.0016042)

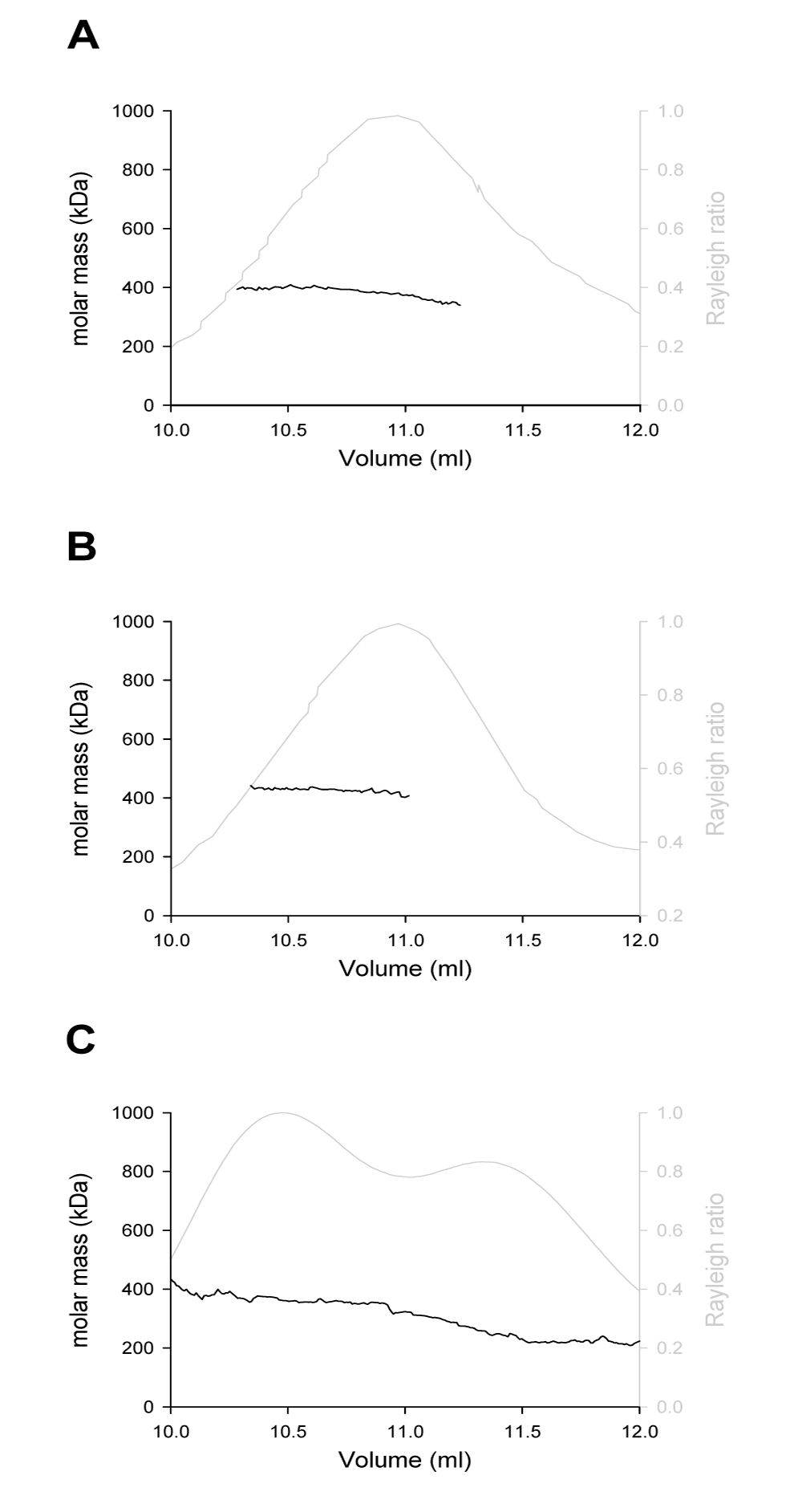

Supplement: Figure S1 — Size exclusion chromatography coupled to static light scattering analysis of Pdx1 wild type, R288A and K187A. Prior to multi-angle light scattering (MALS) measurements, size exclusion chromatography (SEC) was performed using a Superdex 200 10/300 column in 50 mM potassium phosphate buffer, pH 7.5, containing 50 mM potassium chloride using 1 mg/ml of enzyme. The combination of SEC and MALS reveal a predominant peak corresponding to the dodecameric form of Pdx1 (A) wild type or (B) Pdx1 R288A whereas (C) Pdx1K187A exhibits a dodecamer:hexamer equilibrium. The weight average molecular mass Mw mass (black line) and the Rayleigh ratio (gray line) were plotted in an elution volume dependent manner. (TIF) [file pone.0016042.s001.tif]

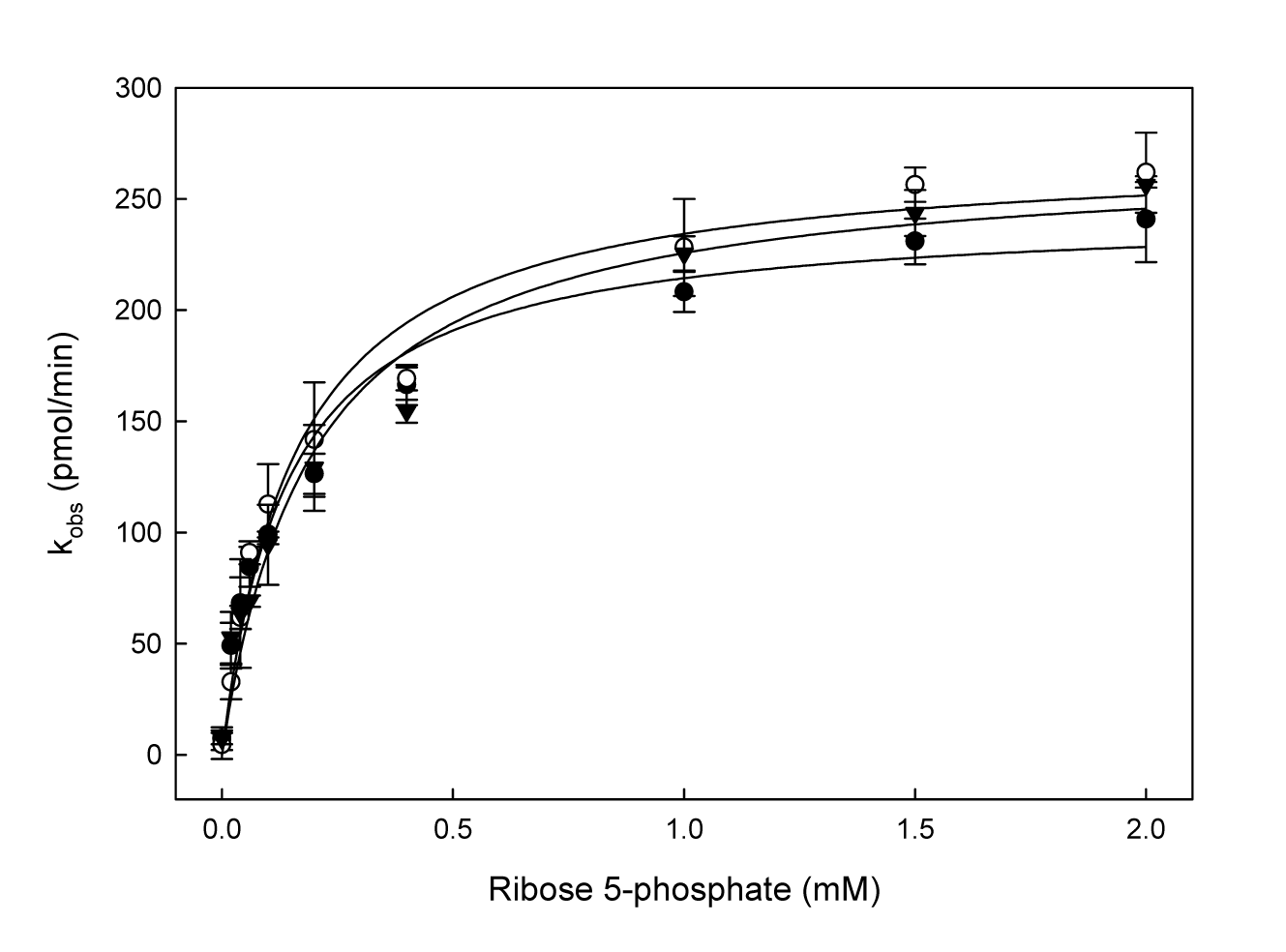

Supplement: Figure S2 — Steady state kinetics for the release of inorganic phosphate from Pdx1 wild type (○), R288A (•) or R288K (▾). The rate of inorganic phosphate release was monitored employing a coupled enzymatic assay. The reaction was carried out in 50 mM Tris-Cl pH 7.5, containing 20 mM magnesium chloride, employing Pdx1 wild type or muteins (20 µM), Pdx2 (20 µM), glutamine (10 mM), 2-amino-6-mercapto-7-methyl-purine (0.2 mM) and purine nucleoside phosphorylase (1.5 U) as a function of the concentration of R5P. (TIF) [file pone.0016042.s002.tif]
