# Supplementary material for: It Takes Two to Tango: Defining an Essential Second Active Site in Pyridoxal 5′-Phosphate Synthase
Source: PLoS One. 2011 Jan 21;6(1):e16042. doi: 10.1371/journal.pone.0016042 (PMC3024981; doi:10.1371/journal.pone.0016042)
Supplement: Table S1 — (DOC) [file pone.0016042.s003.doc]

**Table S1:**

| Table S1: Summary of inorganic phosphate release kinetic constants^a^ | | | |
| --- | --- | --- | --- |
|  | K_M_ R5P | k_cat_ | k_cat_/K_M_ |
| P_i_ release | (mM) | (min^-1^) | (min^-1^.mM^-1^) |
| Pdx1 wild type | 0.16 ± 0.02 | 0.067 ± 0.003 | 0.43 |
| Pdx1 R288K | 0.19 ± 0.04 | 0.067 ± 0.004 | 0.35 |
| Pdx1 R288A | 0.14 ± 0.02 | 0.061 ± 0.003 | 0.44 |
| ^a^Assays were performed at 37°C in 50 mM Tris-Cl, pH 7.5 | | | |
