# Supplementary material for: It Takes Two to Tango: Defining an Essential Second Active Site in Pyridoxal 5′-Phosphate Synthase
Source: PLoS One. 2011 Jan 21;6(1):e16042. doi: 10.1371/journal.pone.0016042 (PMC3024981; doi:10.1371/journal.pone.0016042)
Supplement: Table S2 — (DOC) [file pone.0016042.s004.doc]

**Table S2**

| Table S2: Oligonucleotide sequences used for site directed mutagenesis of *Bs*Pdx1 wild type | |
| --- | --- |
| Name | Sequence 5′-3′ |
| R288A | CTTACTTCCAGAACAGGCTATGCAAGAACGC |
| R288K | CTCAAACTTACTTCCAGAACAGAAGATGCAAGAACGCGGCTGG |
| H115A | CTGACGAAGAATTTGCTTTAAATAAAAATG |
| R137A | CTTGGTGAAGCAACAGCCCGTATTGCGGAAGGTGC |
| R138A | GAAGCAACACGCGCTATTGCGGAAGG |
| R137A/R138A | CTTGGTGAAGCAACAGCCGCTATTGCGGAAGGTGC |
| H115A/R138A | GAAGCAACACGCGCTATTGCGGAAGG |
| K187A | CTAATGACAGAAGCGGCAAACCTAGGTGCTCC |
| E105A | ATTGATGAAAGTGCAGTTCTGACGCC |
| E105D | ATTGATGAAAGTGACGTTCTGACGCC |
